# Small‐Molecule Targeting MuRF1 Protects Against Denervation‐Induced Diaphragmatic Dysfunction: Underlying Molecular Mechanisms

**DOI:** 10.1002/jcsm.70119

**Published:** 2025-11-16

**Authors:** Fernando Ribeiro, Paulo R. Jannig, Siegfried Labeit, Anselmo S. Moriscot

**Affiliations:** ^1^ Department of Anatomy, Institute of Biomedical Sciences University of São Paulo São Paulo Brazil; ^2^ Department of Physiology and Pharmacology, Biomedicum Karolinska Institutet Stockholm Sweden; ^3^ Department for Integrative Pathophysiology, DZHK Partner Site Mannheim‐Heidelberg, Medical Faculty Mannheim University of Heidelberg Mannheim Germany; ^4^ Myomedix GmbH Neckargemünd Germany

**Keywords:** mechanical unloading, MyoMed‐205, PI3K‐Akt–mTOR pathway, skeletal muscle, TRIM63, ubiquitin‐proteasome system, unilateral diaphragm denervation

## Abstract

**Background:**

Mechanical inactivity rapidly induces diaphragm muscle fibres' contractile dysfunction and atrophy. Diaphragm weakness can impair respiratory function, quality of life and increase risks of morbidity and mortality. Muscle RING‐finger protein‐1 (MuRF1) expression is upregulated during denervation and muscle inactivity and is known to target key muscle proteins for degradation. We previously reported that the small‐molecule targeting MuRF1 (MyoMed‐205) protects against diaphragm contractile dysfunction and atrophy after 12 h of unilateral diaphragm denervation (UDD) in rats. In this study, we investigated the mechanisms by which MyoMed‐205 protects the diaphragm structure and function during early UDD in rats.

**Methods:**

Male Wistar rats were subjected to unilateral diaphragm denervation (UDD) for 12 h. Immediately after UDD, rats received either a placebo (vehicle) or small‐molecule targeting MuRF1 (MyoMed‐205, 50 mg/kg bw), and outcomes were compared with sham‐operated controls. Diaphragm was used for histological, morphometric, transcriptomic (RNA‐seq) and protein content (Western blot) analysis.

**Results:**

UDD induced diaphragm slow‐ (Type I: *p* = 0.03) and fast‐twitch (Type IIa: *p* = 0.04; Type IIb/x: *p* = 0.02) fibres atrophy after 12 h, which was prevented by MyoMed‐205 (*p* < 0.05). Mechanistically, UDD perturbed mechanisms involved with myofibre ultrastructure and contractility, mitochondrial function, proteolysis and tissue remodelling in the diaphragm. MyoMed‐205 enhanced the activation of mechanisms required for sarcomere integrity, calcium handling, antioxidant defence, chaperone‐mediated unfolded protein response and muscle growth. MyoMed‐205 also mitigated intramuscular fat deposition and pro‐fibrotic responses triggered by UDD.

**Conclusions:**

Small‐molecule targeting MuRF1 (MyoMed‐205) protects against diaphragm muscle contractile dysfunction and atrophy after 12 h of UDD. Herein, we demonstrate that this protective effect involved augmented activation of signalling pathways controlling muscle structure and function, chaperone‐mediated unfolded protein and muscle growth, while mitigating intramuscular fat deposition and pro‐fibrotic responses triggered by UDD at the transcriptional and/or protein level.

## 1 Introduction

Breathing is essential for life. This key biological process is regulated by a complex neuromuscular network generating intrathoracic pressures for lung ventilation and gas exchange. The diaphragm functions as the primary inspiratory pump while contributing to other physiological processes (e.g., visceral organization, coughing and venous hemodynamics). Because of unique evolutionary adaptations and high lifelong contractile activity, the diaphragm might respond distinctly to mechanical inactivity compared to other skeletal muscles [1]. Clinical conditions such as phrenic nerve injury (e.g., thoracic surgery, trauma) and prolonged mechanical ventilation cause profound diaphragm structural and functional changes. Resulting diaphragm muscle weakness and wasting can impair respiratory capacity, exercise tolerance, quality of life and increase mortality [2].

Understanding how mechanical activity affects diaphragm muscle structure and function has special clinical relevance (for review, see [3, 4]). The unilateral diaphragm denervation (UDD) model has been used to investigate the diaphragm adaptations to short‐ and long‐term periods of absent innervation and contractile function. Even short periods (e.g., < 24 h) of inactivity following UDD significantly decrease diaphragm force‐generation capacity [5, 6]. Within 24 h, contractile function loss can accompany slow‐ and fast‐twitch fibres atrophy [6, 7], although other findings suggest that fibre size may be less affected initially, especially in slow‐twitch fibres [5]. Adaptive responses triggered by UDD include diaphragm ultrastructural changes, myofibrillar protein loss, altered protein balance, increased activation of proteolytic pathways, apoptosis and muscle tissue remodelling [6, 8, 9, 10, 11, 12, 13].

Since its discovery in the early 2000s, muscle RING‐finger protein 1 (MuRF1) has been recognized as a key regulator of skeletal muscle mass and function [14, 15]. This muscle‐specific E3 ligase targets protein clusters controlling muscle contraction, Ca2+ handling, energy metabolism, immune response and protein breakdown [16, 17, 18]. Increased MuRF1 expression/activity occurs in several stress states (e.g., immobilization, denervation, corticosteroids, heart failure, cancer cachexia, aging) and is associated with muscle wasting and weakness, while MuRF1 deletion improves muscle mass and function [19]. Recent advances in small‐molecule MuRF1 inhibitors have shown therapeutic potential across multiple conditions, attenuating muscle wasting in heart failure [20, 21, 22], cancer cachexia [23], corticosteroid‐induced myopathies [20] and diabetes [24]. Notably, MuRF1 deletion or inhibitor protects against early diaphragmatic dysfunction in mechanically ventilated rodents [25, 26], and our group previously demonstrated that small‐molecule MuRF1 targeting (MyoMed‐205) prevents early diaphragm contractile dysfunction and atrophy in rats subjected to UDD [6], suggesting MuRF1 as a promising therapeutic target for counteracting denervation‐induced diaphragm muscle weakness and wasting.

This study aimed to investigate the cellular and molecular mechanisms underlying the small‐molecule targeting (MyoMed205) mediated protection against diaphragm dysfunction and atrophy in rats subjected to 12 h of UDD. We hypothesized that 1) MuRF1 plays a key role in denervation‐induced diaphragmatic dysfunction and atrophy, and 2) MyoMed‐205 differentially regulates signalling pathways controlling diaphragm muscle contractile function, mass and tissue remodelling during denervation stress.

## 2 Material and Methods

### 2.1 Animals

Wistar rats (male, 2–3 months old) were used in this study. The animals were kept in standard cages under controlled environmental conditions (24 ± 1°C, 12 h/12 h light–dark cycle) with access to standard food and water *ad libitum*. This study was approved and followed the institutional guidelines for animal care and use for research (CEUA ICB USP #8728030320 and #5143091020).

### 2.2 Experimental Design

Twenty‐four rats were randomly allocated into three experimental groups (*n* = 8): sham 12 h (sham); unilateral diaphragm denervation (UDD) 12 h + vehicle (DNV + VEH); and UDD 12 h + MyoMed‐205 (DNV + 205). After 12 h, the animals were euthanized under anaesthesia, and the denervated right diaphragm muscle (costal portion) was harvested, quickly snap‐frozen in 2‐methylbutane chilled in liquid nitrogen and stored at −80°C until further analysis. Diaphragm samples used in this study originate from our previous work [6] (for further details, see supplementary information).

### 2.3 MyoMed‐205 Formulation and Delivery

A detailed description of MuRF1 inhibitor (MyoMed‐205, Myomedix GmbH, Germany) formulation for the in vivo study is provided in the supplementary information. Briefly, the compound was freshly dissolved in an 8 mL vehicle solution (DMSO‐PEG400‐Saline 0.9%). The delivery of either vehicle (VEH) or MyoMed‐205 (205) treatments was managed in four split doses (2 mL/dose) administered every 3‐h interval over the 12‐h experimental period via the caudal vein. Treatments started immediately following the confirmation of unilateral diaphragm paralysis (for further details, see supplementary information).

### 2.4 Unilateral Diaphragm Denervation

Animals were initially sedated and anaesthetized via intraperitoneal administration of acepromazine (2.5 mg/kg bw), followed after 30 min by the administration of ketamine (100 mg/kg bw) and xylazine (5 mg/kg bw). After reaching the surgical plane, the right phrenic nerve was exposed and transected at the rat's lower neck region. The absence of right diaphragm dome contractile activity was used to confirm the efficacy of the denervation. Then, the surgical wound was sutured and managed with antiseptics. During the postoperative period, animals were allowed to recover from anaesthesia in individual cages with ad libitum access to food chow and water. (for further details, see supplementary information).

### 2.5 Diaphragm Muscle Fibre Typing and Size Analysis

Cryosections (8 μm thick) from the right costal diaphragm muscle were prepared using a cryostat at −20°C (Leica CM1850, Germany). Muscle fibre typing (MyHC I, IIA, IIB/X) was performed via immunofluorescence assay (see antibodies in Table S1). Photomicrographs were acquired using an Axio Scope A1 microscope (Zeiss, Germany). Diaphragm fibres' type distribution and cross‐sectional area (CSA) were assessed with ImageJ (Fiji, NIH, USA). Representative muscle fibre type distribution and CSA values were determined as the average from 300 measurements per sample of previously unanalyzed muscle fibres (for further details, see supplementary information).

### 2.6 Oil Red O Staining

Diaphragm muscle cryosections were stained with Oil Red O (ORO) to assess the intramuscular lipid content. Photomicrographs were acquired using an Axio Scope A1 microscope (Zeiss, Germany). The representative number of positive ORO (ORO+) muscle cells and intracellular lipid content were determined based on measurements of 300 fibres per sample (for further details, see supplementary information).

### 2.7 Transcriptomic Profiling (RNA‐Seq)

Total RNA was extracted from the diaphragm using TRIzol (Life Technologies, Carlsbad, CA). RNA integrity was analyzed using Bioanalyzer 2100 (Agilent, Santa Clara, CA, USA) (Table S2). Four samples of each experimental group with the highest purity and integrity levels were selected for global gene expression analysis (Figure S1). Approximately 1 μg of sample total RNA was used for cDNA library preparation performed according to the Illumina mRNA stranded poly‐A tail enrichment Kit manufacturer's instructions (Illumina, San Diego, CA, USA). RNA sequencing was carried out using the Illumina NextSeq 2000 (NextSeq 2x100pb, 20 million paired‐end reads depth). These procedures were performed by NGS Soluções Genômicas (Piracicaba, Brazil).

For bioinformatic analysis, quality control of raw reads was performed using the FastQC toolkit (Babraham Bioinformatics). The reads were then aligned to the mRatBN7.2 rat genome using STAR. Feature count was performed using featureCounts, and differential expression analyses were generated in R using the DESeq2 package with adaptive log‐fold change shrinkage estimator from the ashr package Pathway analysis was carried out by Gene Set Enrichment Analysis (GSEA) using a preranked list of genes by log2Fold‐Change and Molecular Signatures Database, and hallmark pathways gene sets for pathway analysis. Transcriptome‐related raw files and data can be found in the Gene Expression Omnibus (GEO) repository (GSE305304) (for further details, see supplementary information).

### 2.8 Western Blotting

Diaphragm samples were powdered in a liquid nitrogen‐chilled mortar and homogenized in RIPA buffer containing protease and phosphatase inhibitors (ThermoFisher #78445). Homogenates were centrifuged and collected and stored at −80°C until further analysis. Protein concentration was determined by the Bradford method with bovine serum albumin (BSA) as the standard. Protein was loaded onto polyacrylamide gel (SDS‐PAGE), separated via electrophoresis (100 V) and transferred to polyvinylidene difluoride (PVDF) membrane (Thermo Fisher Scientific, #88518, Rockford, IL, USA) using a wet‐transfer system (100 V, 350 mA, for 120 min). Ponceau red staining was used to assess protein transfer efficacy. Membranes were incubated with primary and secondary antibodies (Table S1). Membranes were incubated with ECL substrate (Immobilon Forte, Millipore #WBLUF0500) for 5 min before signal detection using a C‐digit blot scanner (Li‐Cor, USA) for chemiluminescent detection or with BCIP/NBT substrate (Sigma #B1911) for colorimetric detection. Alpha‐tubulin was used as a loading control for data normalization, and densitometry analysis was performed using ImageStudio software (for further details, see supplementary information).

### 2.9 Statistical Analysis

Data analysis was performed using Excel, Prism and R. For the analysis of the transcriptomic data, refer to the respective section above and supplementary information. For the analysis of histological, morphometric and protein expression levels, the Shapiro–Wilk test was used to assess data normality. One‐way ANOVA followed by Tukey's post hoc test, or the Kruskal–Wallis test followed by Dunn's test, was used for multiple comparisons between groups, for parametric and nonparametric data, respectively. Data are presented as mean ± standard deviation, and statistical significance was accepted as *p* < 0.05.

## 3 Results

### 3.1 Transcriptomic Profiling Reveals Mechanisms Underlying MyoMed‐205–Mediated Protection Against Denervation‐Induced Diaphragmatic Dysfunction and Atrophy

**FIGURE 1:**
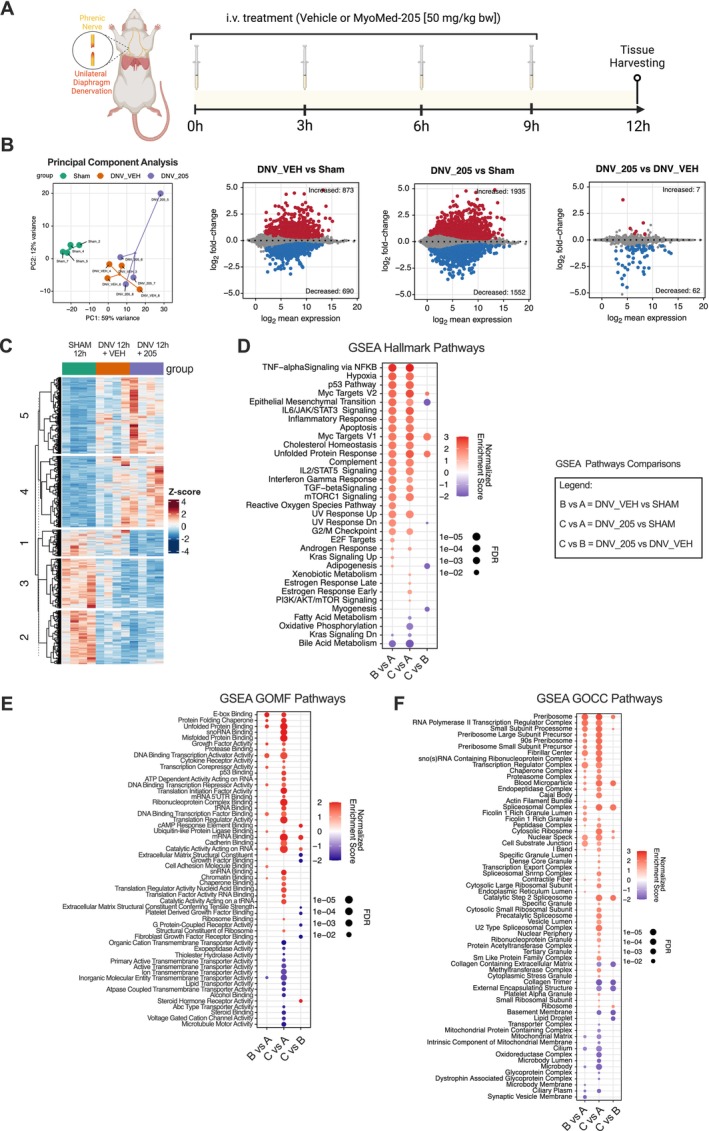
Transcriptomic profiling of the diaphragm following 12 h of UDD and identification of pathways responsive to MyoMed‐205. (A) Schematic illustration of the experimental design used to investigate the effect of small‐molecule targeting MuRF1 (MyoMed‐205, 50 mg/kg bw, intravenous delivery) upon early UDD pathophysiology in rats. (B) Principal component analysis for RNA‐seq of diaphragm muscle and identification of differentially expressed genes (DEGs, *p*_adj_ < 0.05) across the distinct experimental conditions (*n* = 4 per group). Upregulated genes are represented in red, while downregulated genes are represented in blue. (C) Hierarchical clustered heat map highlights five clusters of DEGs identified in response to UDD and MyoMed‐205 compared with the control. (D) GSEA reveals the hallmark pathways responsive to UDD and the impacts of MyoMed‐205. Additionally, GSEA indicates the altered (E) molecular functions (GOMF) and (F) cellular compartments (GOCC) of the diaphragm muscle in response to UDD and MyoMed‐205 compared with controls. FDR, false discovery rate; GSEA, Gene Set Enrichment Analysis; *p*_adj_, adjusted *p* value; UDD, unilateral diaphragm denervation.

To investigate how denervation might impair diaphragm muscle structure and contractile function, as reported previously [6], we performed a global gene expression analysis of the rat's diaphragm following 12 h of UDD. Additionally, we compared the transcriptomes of MyoMed‐205–treated animals with untreated animals to investigate the mechanisms underlying the MyoMed‐205–mediated protective effects. For this purpose, animals were treated either with vehicle (DNV_VEH) or MyoMed‐205 (DNV_205), and results were compared with sham controls (Figure 1A).

Principal component analysis (PCA) demonstrated significant transcriptomic alterations in DNV_VEH compared with sham. On the other hand, MyoMed‐205 caused no evident changes in the clustering pattern compared with DNV_VEH (Figure 1B). DESeq2 analysis was performed to identify differentially expressed genes (DEGs) for each condition (*p*_adj_ < 0.05). We detected 1563 DEGs in DNV_VEH (873 upregulated, 690 downregulated) and 3487 DEGs in DNV_205 (1935 upregulated, 1552 downregulated), compared with sham. We found 69 DEGs particularly responsive to MyoMed‐205 treatment compared with DNV_VEH (Figure 1B), of which 7 were upregulated and 62 downregulated (see Table 1). Hierarchical clustering analysis revealed five gene clusters regulated by UDD and MyoMed‐205 (Figure 1C).

**TABLE 1:** Differentially expressed genes altered by MyoMed‐205 treatment compared with vehicle after 12 h of UDD.

ENSEMBL ID	Gene symbol	Gene biotype	Chromossome	Base mean	Log2 fold change	lfcSE	*p*	*p*_adj_
UP								
ENSRNOG00000016437	Tm4sf4	Protein_coding	2	17.6122429	3.789838599	1.203520958	1.75071E‐06	0.001204378
ENSRNOG00000005964	Nr4a3	Protein_coding	5	283.3148153	1.606680313	0.65174188	8.04E‐06	0.004055837
ENSRNOG00000042717	Ciart	Protein_coding	2	47.50701374	1.087136024	0.241878824	2.134E‐07	0.000240969
ENSRNOG00000012410	S100a1	Protein_coding	2	106.230202	0.806337451	0.179253103	6.33191E‐07	0.000560089
ENSRNOG00000026519	Pkm‐ps20	Pseudogene	20	87.81533493	0.596757872	0.391477297	0.000224519	0.049981909
ENSRNOG00000011262	Bicra	Protein_coding	1	352.0030887	0.566342079	0.18605263	1.60898E‐05	0.00658292
ENSRNOG00000038212	Socs6	Protein_coding	18	357.1032721	0.504293982	0.239060527	8.12826E‐05	0.023662613
DOWN								
ENSRNOG00000008310	Mpo	Protein_coding	10	30.68728358	−4.477626874	0.86497719	1.23056E‐08	3.10471E‐05
ENSRNOG00000021207	Lgals12	Protein_coding	1	16.63787864	−4.226564414	1.462497668	1.63231E‐06	0.001176662
ENSRNOG00000002834	Klb	Protein_coding	14	15.17946604	−3.653141743	0.722901877	3.1499E‐08	5.96041E‐05
ENSRNOG00000012674	Adrb3	Protein_coding	16	20.96107168	−3.344566786	0.622993833	7.83432E‐09	2.37192E‐05
ENSRNOG00000028616	Pck1	Protein_coding	3	316.026661	−3.239192936	0.449947119	1.29121E‐13	1.95463E‐09
ENSRNOG00000046858	Arxes2	Protein_coding	X	8.819805912	−3.068825083	1.295518258	6.734E‐06	0.003533777
ENSRNOG00000046858	Arxes1	Protein_coding	X	8.819805912	−3.068825083	1.295518258	6.734E‐06	0.003533777
ENSRNOG00000015086	Plin1	Protein_coding	1	284.6420376	−3.002527864	0.639015708	8.30602E‐08	0.000125737
ENSRNOG00000049351	Nat8l	Protein_coding	14	33.73179932	−2.809515359	0.684655737	3.75906E‐07	0.000355654
ENSRNOG00000001821	Adipoq	Protein_coding	11	607.3094283	−2.658603085	0.609454255	1.55575E‐07	0.000196258
ENSRNOG00000009153	Cidec	Protein_coding	4	319.8284686	−2.639766654	0.454193794	2.75621E‐10	1.90248E‐06
ENSRNOG00000011892	Slc36a2	Protein_coding	10	23.77518803	−2.610864966	0.971462989	4.89381E‐06	0.00284933
ENSRNOG00000001295	S100b	Protein_coding	20	138.0192366	−2.598281841	0.52717817	1.73533E‐08	3.75278E‐05
ENSRNOG00000013097	C6h14orf180	Protein_coding	6	16.4152481	−2.410504532	1.098357941	1.05224E‐05	0.004704953
ENSRNOG00000005792	Ankef1	Protein_coding	3	40.92764889	−2.287926971	0.871341313	5.35876E‐06	0.00300448
ENSRNOG00000004828	Acvr1c	Protein_coding	3	9.367554476	−2.259186154	1.653522054	3.58143E‐05	0.012908502
ENSRNOG00000009079	Prkar2b	Protein_coding	6	186.3610277	−2.151611484	0.606796375	7.87982E‐07	0.000627814
ENSRNOG00000003442	Adora1	Protein_coding	13	68.96023553	−2.116664501	0.48780618	5.4613E‐08	9.18591E‐05
ENSRNOG00000001001	Retn	Protein_coding	12	176.1481687	−2.097250535	0.583972591	6.6598E‐07	0.000560089
ENSRNOG00000012278	Fgf10	Protein_coding	2	7.061425597	−1.811214281	1.476219539	5.76646E‐05	0.01857291
ENSRNOG00000015071	Zim1	Protein_coding	1	72.74520752	−1.725675207	0.546423231	1.82988E‐06	0.001204378
ENSRNOG00000033564	Cfd	Protein_coding	7	341.3879947	−1.618698388	0.396036993	1.0744E‐07	0.000147856
ENSRNOG00000008956	Cdkn2c	Protein_coding	5	39.78068637	−1.571364197	0.400702292	2.22854E‐07	0.000240969
ENSRNOG00000022946	Slc22a3	Protein_coding	1	35.26224121	−1.546451726	0.647987749	9.70743E‐06	0.00459222
ENSRNOG00000000158	Cdo1	Protein_coding	18	475.4382482	−1.494996513	0.282306833	3.77026E‐10	1.90248E‐06
ENSRNOG00000011417	Pde3b	Protein_coding	1	70.10126466	−1.275540592	0.599106026	2.08486E‐05	0.008305415
ENSRNOG00000003357	Col3a1	Protein_coding	9	12381.13309	−1.269296892	0.412765196	4.54009E‐06	0.002830397
ENSRNOG00000022268	Pnpla3	Protein_coding	7	66.42415379	−1.226076719	1.362324254	0.000145765	0.036173668
ENSRNOG00000018951	Col4a5	Protein_coding	X	246.4573269	−1.172681715	0.271286522	2.56263E‐07	0.000258621
ENSRNOG00000012840	Sparc	Protein_coding	10	9459.030856	−1.145582612	0.219721436	5.42177E‐09	2.05187E‐05
ENSRNOG00000016681	Mc2r	Protein_coding	18	6.745401153	−1.044223744	1.376734915	0.000215527	0.049433986
ENSRNOG00000068207	ENSRNOG00000068207	lncRNA	2	71.69491139	−1.040907016	0.337064148	8.52713E‐06	0.004163992
ENSRNOG00000059894	Hmmr	Protein_coding	10	14.3711061	−1.01971652	0.746399567	9.21002E‐05	0.024896662
ENSRNOG00000019265	Pcdh12	Protein_coding	18	97.81810717	−1.00564771	0.288661155	4.67432E‐06	0.002830397
ENSRNOG00000029911	Cilp	Protein_coding	8	350.9021455	−0.996386598	0.302124862	6.76969E‐06	0.003533777
ENSRNOG00000003897	Col1a1	Protein_coding	10	5473.455718	−0.972013538	0.595029291	7.39457E‐05	0.022035732
ENSRNOG00000060381	Col15a1	Protein_coding	5	1094.025407	−0.970012729	0.238654195	1.41264E‐06	0.001069228
ENSRNOG00000005998	Smoc1	Protein_coding	6	43.56168462	−0.961648386	0.625367727	8.46648E‐05	0.024182175
ENSRNOG00000049614	Itih5	Protein_coding	17	259.6072555	−0.954845167	0.321664858	1.3057E‐05	0.005647348
ENSRNOG00000022239	Trarg1	Protein_coding	10	215.502458	−0.946272472	0.508271459	6.02012E‐05	0.018985952
ENSRNOG00000048924	Islr	Protein_coding	8	96.75848625	−0.932560953	0.475548876	5.52929E‐05	0.018196186
ENSRNOG00000014791	Peg3	Protein_coding	1	1008.607567	−0.929639141	0.296025766	1.05673E‐05	0.004704953
ENSRNOG00000029465	Slc26a10	Protein_coding	7	318.7370695	−0.92657907	0.520335672	6.97254E‐05	0.021540881
ENSRNOG00000012660	Postn	Protein_coding	2	407.3694786	−0.926272454	0.581975445	8.67208E‐05	0.024310733
ENSRNOG00000017406	Atrnl1	Protein_coding	1	86.66961975	−0.922922419	0.351946922	2.36397E‐05	0.009175822
ENSRNOG00000007059	Atp1b4	Protein_coding	X	126.8800026	−0.903643521	0.647470525	0.000113779	0.030217437
ENSRNOG00000020525	Col5a3	Protein_coding	8	282.2641751	−0.849485674	0.381327901	4.85208E‐05	0.01632241
ENSRNOG00000003869	Sod3	Protein_coding	14	344.0210729	−0.841802222	0.322322641	2.88874E‐05	0.010665786
ENSRNOG00000026114	Ap5b1	Protein_coding	1	71.33655764	−0.825421112	0.301195018	2.46814E‐05	0.009340692
ENSRNOG00000011921	Dusp4	Protein_coding	16	58.03429501	−0.820283796	0.576863771	0.000137595	0.0359122
ENSRNOG00000020546	Lipe	Protein_coding	1	652.1235112	−0.778821842	0.383241395	7.42385E‐05	0.022035732
ENSRNOG00000047940	Mmp28	Protein_coding	10	28.56867425	−0.761946897	0.618130313	0.000194535	0.045651031
ENSRNOG00000018865	Adamts12	Protein_coding	2	166.9839783	−0.71216855	0.432342073	0.000144709	0.036173668
ENSRNOG00000007370	Rnf144a	Protein_coding	6	34.97523865	−0.680235649	0.414843868	0.000158104	0.038602792
ENSRNOG00000003736	Col5a2	Protein_coding	9	961.2698157	−0.677069523	0.265562027	4.01151E‐05	0.014122369
ENSRNOG00000007300	C1qtnf6	Protein_coding	7	61.36282397	−0.674758918	0.492847777	0.000223652	0.049981909
ENSRNOG00000005323	Syne2	Protein_coding	6	714.2872969	−0.592748527	0.234918406	4.19457E‐05	0.014431222
ENSRNOG00000021437	Hspg2	Protein_coding	5	3301.965162	−0.586663851	0.187505171	1.43459E‐05	0.006032463
ENSRNOG00000043192	Hacd1	Protein_coding	17	302.3667613	−0.574867934	0.337959209	0.00017446	0.041920198
ENSRNOG00000016281	Col4a1	Protein_coding	16	4047.849222	−0.560805627	0.34135596	0.000196018	0.045651031
ENSRNOG00000023400	Dtx3l	Protein_coding	11	119.2932824	−0.555706162	0.303233193	0.000140476	0.03604287
ENSRNOG00000021237	Dnaaf9	Protein_coding	3	161.2939517	−0.521175226	0.25306968	9.13532E‐05	0.024896662

From the 69 DEGs identified in DNV_205 versus DNV_VEH listed above, 7 DEGs were upregulated, while other 62 DEGs were downregulated in DNV_205 compared with DNV_VEH group. The DEGs are presented as UP and DOWN categories and ranked according to the highest to lowest fold change value.

Abbreviations: DOWN, downregulated; lfcSE, log fold change standard error; *p*_adj_, adjusted *p* value; UP, upregulated.

Gene Set Enrichment Analysis (GSEA) was subsequently employed to identify biological pathways modulated by UDD and MyoMed‐205, including hallmark pathways (Figure 1D), molecular functions (Figure 1E) and cellular compartments (Figure 1F). UDD significantly altered the expression of genes associated with muscle contraction, mitochondrial structure/function, inflammatory responses, proteolysis, apoptosis and muscle tissue remodelling, compared with sham. Interestingly, MyoMed‐205 upregulated pathways involved with antioxidant response, chaperone‐mediated unfolded protein response (UPR), ribosome biogenesis, protein synthesis and cell growth. Conversely, MyoMed‐205 downregulated genes associated with adipogenesis and extracellular matrix collagen deposition compared with DNV_VEH and sham groups (Figure 1D–F).

### 3.2 Effects of MyoMed‐205 Upon Mechanisms That Control Skeletal Muscle Structure and Contractile Function

**FIGURE 2:**
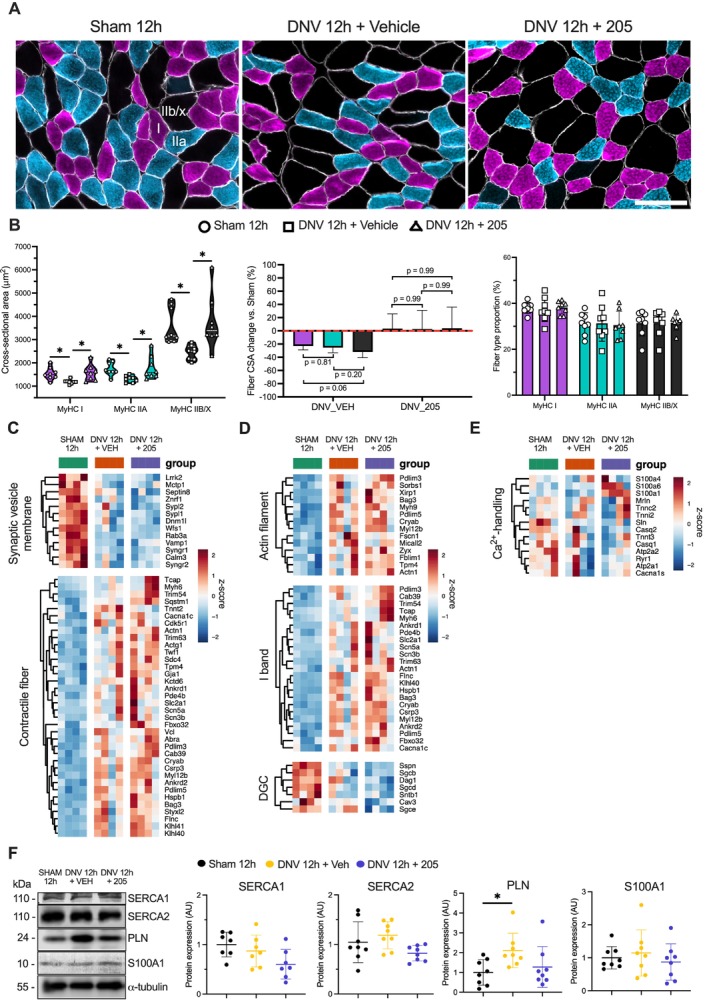
MyoMed‐205 protects against diaphragm fibre atrophy induced by 12 h of unilateral diaphragm denervation (UDD). (A) Representative photomicrographs of diaphragm muscle immunolabelled with antibodies against the myosin heavy chain isoforms, Type I (violet), Type IIa (light blue), Type IIb/x (black) and dystrophin (white) for morphometric analysis by fibre type. Scale bar = 100 μm. (B) Fibres' CSA, relative CSA percentual change compared with sham‐controls and relative percentual distribution by fibre type. Data are presented as mean and standard deviation (*n* = 8). One‐way ANOVA followed by Tukey's post hoc test was used for statistical comparisons among the groups. *p < 0.05. (C) Heat map of genes linked to muscle contraction (i.e., synaptic vesicle membrane and contractile fibre). (D) Heat map of genes linked to sarcomere structure and function (i.e., actin filament, I band and DCG). (F) Protein levels of calcium‐handling markers were assessed by Western blotting. Data are presented as mean and standard deviation of the fold change relative to the control group (*n* = 7–8). One‐way ANOVA followed by Tukey's post hoc test was used for statistical comparisons among the groups. *p < 0.05. CSA, cross‐sectional area; DCG, dystrophin glycoprotein complex; MyHC, myosin heavy chain.

We previously reported that 12 h of UDD promotes muscle fibres' contractile dysfunction and atrophy [6]. Herein, our histomorphometric analysis confirmed a significant decrease in diaphragm fibre size across all types (sham vs. DNV_VEH vs. DNV_205, mean ± SD—Type I: 1537 ± 269 vs. 1184 ± 91 vs. 1588 ± 342; Type IIa: 1750 ± 295 vs. 1307 ± 139 vs. 1795 ± 497; Type IIb/x: 3601 ± 788 vs. 2449 ± 308 vs. 3741 ± 1153 μm^2^, *p* < 0.05, *n* = 8) following 12 h of UDD (Type I: *p* = 0.03; Type IIa: *p* = 0.04; and Type IIb/x: *p* = 0.02) compared with sham. UDD caused comparable atrophy levels among the fibre types, despite a trend indicating a noticeable effect in fast‐twitch Type IIb/x fibres compared with slow‐twitch Type I fibres (*p* = 0.06). Fibre type relative distribution remained unchanged (Figure 2A,B). MyoMed‐205 significantly protected against UDD‐associated fibre atrophy (*p* < 0.05, *n* = 8), without showing fibre type‐specific effect or affecting relative distribution (Figure 2A,B).

To gain mechanistic insights into the protective effects of MyoMed‐205, we examined the expression levels of genes and proteins involved in the control of muscle structure and contraction. Significant changes in genes regulating synaptic vesicle membrane and muscle fibre structure/function were observed following 12 h of UDD (Figure 2C). Notably, MyoMed‐205 selectively enhanced the expression of genes involved with sarcomere organization and integrity, Z‐disk structure, mechanotransduction and intercellular communication (e.g., Tcap, Actn1, Pdlim3, Cryab, Ankrd1, Zyx, Sdc4, Sorbs1, Tpm4, Bag3 and Gja1) (Figure 2C,D).

Furthermore, UDD affected calcium handling pathways as indicated by downregulation of key calcium handling genes (e.g., RyR1, SERCA1 and SERCA2) (Figure 2E) and increased phospholamban protein levels compared with sham (Figure 2F). MyoMed‐205 enhanced S100A1 gene expression, a calcium‐binding protein that positively regulates the activity of calcium channels, concomitantly with suppression of denervation‐induced phospholamban protein expression (Figure 2E–F).

### 3.3 UDD Negatively Affects the Transcription of Genes Associated With Mitochondrial Dysfunction and Oxidative Stress, While MyoMed‐205 Enhances the Transcription of Antioxidant Factors

**FIGURE 3:**
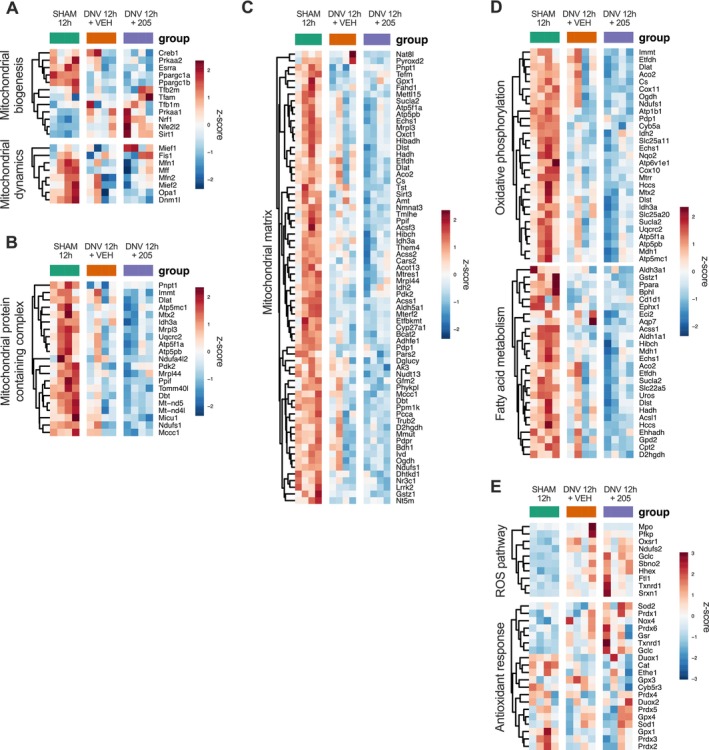
Unilateral diaphragm denervation enhances transcriptional activation of pathways associated with mitochondrial dysfunction and oxidative stress, while MyoMed‐205 upregulates the gene expression of antioxidant factors. (A) Heat map of genes involved in the control of mitochondrial biogenesis and dynamics. (B–C) Heat map of genes associated with mitochondrial structural and functional complexes. (D) Heat map of genes linked to mitochondrial energy production and metabolism. (E) Heat map of genes involved with ROS production and antioxidant response. ROS, reactive oxygen species; UDD, unilateral diaphragm denervation.

Mitochondrial structure and function are essential for the maintenance of skeletal muscle function and homeostasis. Therefore, we assessed mitochondrial alterations following 12 h of UDD and MyoMed‐205 in the diaphragm. Following 12 h of UDD, significant changes were observed in the transcription of genes that control mitochondrial structure and function. Key mitochondrial biogenesis regulators (e.g., Ppargc1a) and dynamics mediators (e.g., Opa1, Mfn1, Mfn2) were markedly downregulated (Figure 3A). Genes encoding mitochondrial structural complexes (Figure 3B–C), as well as those regulating energy metabolism via oxidative phosphorylation and fatty acid oxidation (Figure 3D), were also markedly downregulated, further suggesting rapid changes in the expression of genes that regulate mitochondrial dynamics and function. Moreover, we observed increased transcriptional activation of the reactive oxygen species (ROS) pathway (Figure 3E). Interestingly, MyoMed‐205 upregulated the expression of a subset of genes involved with the redox balance and antioxidant response (e.g., Nrf1, Nfe2l2, Sirt1, Sod2, Prdx1, Prdx6 and Gclc) (Figure 3E).

### 3.4 MyoMed‐205 Enhances Activation of Pathways Regulating Ribosome Biogenesis, Protein Synthesis and Cell Growth Under UDD‐Induced Stress

**FIGURE 4:**
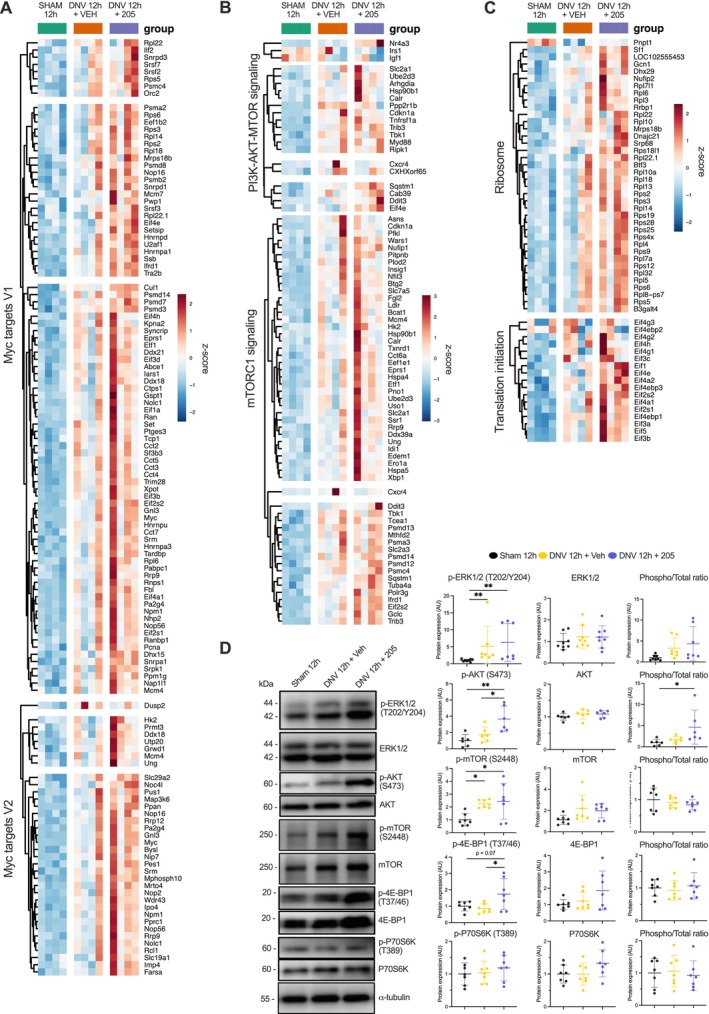
MyoMed‐205 enhances the activation of signalling pathways controlling ribosome biogenesis, protein synthesis and cell growth. (A) Heat map of Myc target genes. (B) Heat map of genes associated with the PI3K‐AKT–mTOR pathway. (C) Heat map of genes controlling ribosome biogenesis and translation initiation. (D) Protein levels of MAPK and PI3K‐Akt–mTOR pathways markers were assessed by Western blotting. Data are presented as mean and standard deviation of the fold change relative to the control group (*n* = 6–7). One‐way ANOVA followed by Tukey's post hoc test was used for statistical comparisons among the groups. *p < 0.05; **p < 0.01.

Denervation can negatively affect muscle mass by altering protein synthesis rate and promoting a negative protein net balance. We evaluated the impacts of UDD and the MyoMed‐205 upon pathways involved with cell growth, proliferation and survival. Notably, Myc target genes (Figure 4A), the KRAS (Figure S3) and mTORC1 signalling pathways were found upregulated after 12 h of UDD (Figure 4B). Strikingly, MyoMed‐205 enhanced RNA processing and transcription (Figure S2), further amplifying these effects by increasing the expression of genes associated with the PI3K‐Akt–mTOR pathway (Figure 4B), ribosome biogenesis, translation initiation (Figure 4C) and androgen response (Figure S3). Consistent with gene expression changes, MyoMed‐205 increased PI3K‐Akt–mTOR pathway activation at the protein level (Figure 4D).

### 3.5 UDD Upregulates Proteolytic Signalling Pathways, While MyoMed‐205 Improves Chaperone‐Mediated Unfolded Protein Response at the Transcriptional Level

**FIGURE 5:**
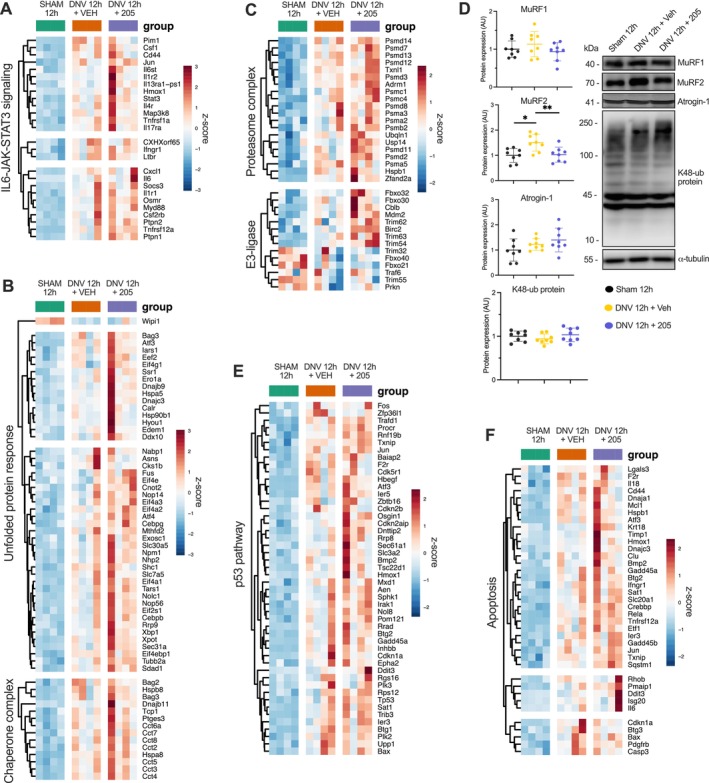
MyoMed‐205 enhances the transcriptional activation of heat shock chaperones and the UPR mechanism in the diaphragm under denervation‐induced cellular stress and catabolic state. (A) Heat map of genes from the IL6‐JAK‐STAT3 pathway. (B) Heat map of genes associated with the chaperone complex and UPR. (C) Heat map of genes linked to the ubiquitin‐proteasome system. (D) Protein levels of ubiquitin‐proteasome pathway markers were assessed by Western blotting. Data are presented as mean and standard deviation of the fold change relative to the control group (*n* = 8). One‐way ANOVA followed by Tukey's post hoc test was used for statistical comparisons among the groups. *p < 0.05; **p < 0.01. (E) Heat maps of genes from the p53 pathway. (F) Heat maps of genes associated with apoptosis. UPR, unfolded protein response.

Denervation can also activate proteolytic pathways contributing to muscle wasting. Thus, we evaluated the effect of UDD and MyoMed‐205 upon pathways associated with muscle protein breakdown and programmed cell death. UDD significantly upregulated distinct proteolytic and stress response pathways, including immune and inflammatory responses (Figure S4), the IL6‐JAK‐STAT3 pathway (Figure 5A), UPR (Figure 5B), ubiquitin‐proteasome pathway (Figure 5C,D), p53 pathway (Figure 5E) and apoptosis (Figure 5F) at the transcriptional level. Remarkably, MyoMed‐205 enhanced the expression of genes mediating chaperone‐mediated UPR (Figure 5B). Noteworthy, we noticed a relatively higher gene expression of E3‐ligases linked to muscle wasting, including Fbxo32 (i.e., Atrogin‐1, log2fold = 0.96 vs. 0.20, values relative to sham; *p*_adj_ = 0.59) and TRIM63 (i.e., MuRF1, log2fold = 2.27 vs. 1.44, values relative to sham; *p*_adj_ = 0.59) in MyoMed‐205 treated animals compared with vehicle, despite not reaching statistical significance (Figure 5C). In line with this, protein levels of these E3‐ligases and K48‐ubiquitinated proteins were not significantly changed, except for MuRF2, a close relative of MuRF1, which was upregulated by UDD but prevented by MyoMed‐205 (Figure 5D).

### 3.6 MyoMed‐205 Mitigates Denervation‐Induced Expression of Genes Involved With Intramuscular Lipid Accumulation and ECM‐Collagen Deposition

Prolonged inactivity can lead to sustained muscle cell stress and damage, triggering apoptosis and tissue remodelling responses such as intramuscular fat deposition and fibrosis. Therefore, we evaluated how UDD and MyoMed‐205 affect these responses in the diaphragm after 12 h.

**FIGURE 6:**
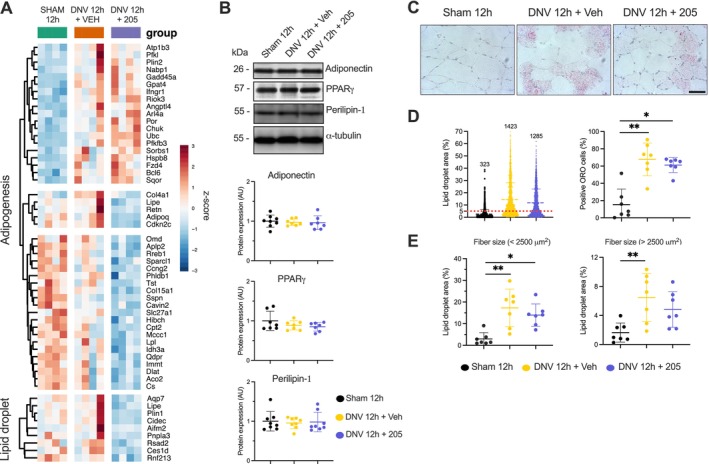
MyoMed‐205 attenuates denervation‐induced intramuscular fat accumulation in the diaphragm by downregulating pro‐adipogenic genes. (A) Heat map of genes related to adipogenesis and intramuscular lipid accumulation. (B) Protein levels of adipogenesis pathway markers were assessed by Western blotting. Data are presented as mean and standard deviation of the fold change relative to the control group (*n* = 8). One‐way ANOVA followed by Tukey's post hoc test was used for statistical comparisons among the groups. (C) Representative photomicrographs of ORO staining of the diaphragm for assessing intramuscular lipid accumulation following 12 h of unilateral diaphragm denervation and the effects of MyoMed‐205. Scale bar = 50 μm. (D) Quantitative analysis of positive ORO muscle cells (i.e., intracellular lipid droplet area > 5% of total muscle fibre area). (E) Mean intracellular lipid droplet area of diaphragm muscle fibres < 2500 μm^2^ (predominantly oxidative metabolism) and fibres > 2500 μm^2^ (predominantly glycolytic metabolism). In total, 300 muscle cells were assessed per sample to estimate the average positive ORO cells and intracellular lipid content. Data are presented as mean and standard deviation (*n* = 7). One‐way ANOVA followed by Tukey's post hoc test was used for statistical comparisons among the groups. *p < 0.05; **p < 0.01. ORO, Oil Red O.

Following UDD, we observed a significant upregulation in adipogenic‐related gene expression compared with sham (Figure 6A), although no changes were detected in adipogenic or lipid droplet‐related proteins within this period (Figure 6B). Oil Red O staining revealed significantly increased ORO‐positive (ORO+) cells and intracellular lipid content across distinct fibre phenotypes (i.e., predominantly oxidative fibres < 2500 μm^2^ and predominantly glycolytic fibres > 2500 μm^2^). Interestingly, MyoMed‐205 downregulated adipogenesis at the transcriptional level (Figure 6A), attenuating intramuscular lipid accumulation triggered by UDD (Figure 6C).

**FIGURE 7:**
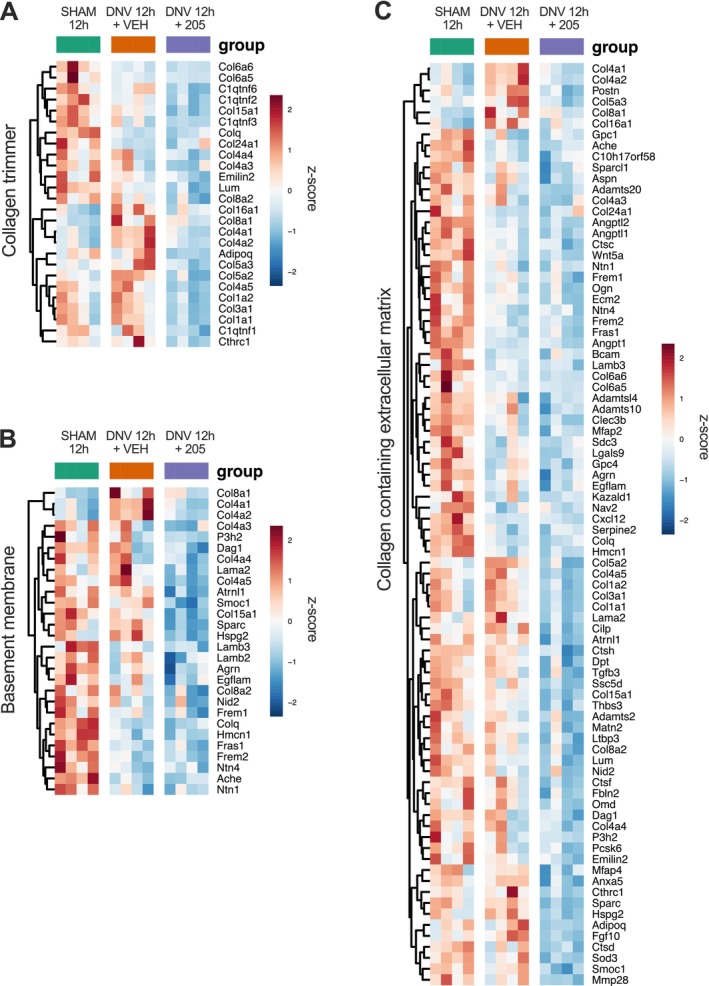
MyoMed‐205 mitigates denervation‐induced upregulation of genes involved with extracellular matrix collagen deposition and fibrosis in the diaphragm at the transcriptional level. (A) Heat map of genes involved in the formation of collagen trimmers. (B) Heat map of genes associated with the basement membrane. (C) Heat map of genes associated with collagen assembly and the extracellular matrix.

Moreover, UDD upregulated the expression of genes associated with the TGF‐β signalling (Figure S5), collagen trimmer (Figure 7A), basement membrane (Figure 7B) and extracellular‐matrix collagen deposition (Figure 7C), compared with sham. On the other hand, MyoMed‐205 downregulated the pro‐fibrotic response at the transcriptional level (Figure 7A–C).

## 4 Discussion

Lack of contractile activity can rapidly induce diaphragm muscle weakness and wasting, contributing to impaired respiratory function, morbidity and mortality. This clinical condition is particularly found in patients undergoing phrenic nerve injury or prolonged mechanical ventilation during intensive care unit (ICU) treatment. Therefore, understanding the mechanisms underlying UDD and how to prevent them by identifying potential novel therapeutic targets is an important, and yet unmet, clinical need. We recently reported that small‐molecule targeting MuRF1 (MyoMed‐205) protects against early diaphragm muscle fibre contractile dysfunction and atrophy in rats undergoing 12 h of UDD [6]. In the present study, we identified molecular mechanisms that are linked to the MyoMed‐205–mediated protection against this loss in fibres' contractile force and size. Briefly, MyoMed‐205 upregulated pathways associated with the following: 1) muscle fibre structure and contractile function; 2) antioxidant defence; 3) chaperone‐mediated UPR; 4) muscle cell growth, while downregulated pathways involved with 5) intramuscular lipid accumulation and 6) ECM collagen deposition at the transcriptional and/or protein level.

Disrupted calcium homeostasis has been reported in response to diaphragm mechanical inactivity, as evidenced by downregulation of key genes like SERCA1, impaired RyR1 activity, decreased Ca2+ sensitivity and sarcoplasmic reticulum (SR) calcium leakage [27]. Consistent with these findings, we observed a rapid downregulation of key genes involved in calcium handling, alongside increased phospholamban protein levels, a negative regulator of SERCA activity. MuRF1 has been reported to directly target proteins involved in calcium handling and muscle contraction [16, 17, 18]. Interestingly, MyoMed‐205 prevented denervation‐induced elevation of phospholamban protein levels, while upregulating S100A1 gene expression, although its protein levels remained unchanged. S100A1 is a calcium‐binding protein that enhances RyR1 and SERCA activity and thus improves excitation‐contraction coupling in striated muscles [28]. S100A1 overexpression has been shown to rescue cardiac function through enhanced Ca^2+^ handling and myofibrillar protein Ca^2+^ responsiveness in heart failure models [29]. These findings suggest that MyoMed‐205 may partially preserve calcium handling homeostasis via upregulation of S100A1 and blockade of phospholamban, likely contributing to preserving diaphragm contractile function, as we previously reported [6].

Myofibrillar protein loss and posttranslational modifications (PTMs) induced by prolonged inactivity can also impair diaphragm force‐generation capacity. ROS‐induced oxidative modifications of muscle proteins have been detected as early as 12 h of UDD. Our data revealed rapid downregulation of key genes for mitochondrial function and augmented activation of oxidative stress pathways, consistent with our previous findings [6]. MuRF1 can directly regulate certain mitochondrial proteins [16, 17, 18]. Here, MyoMed‐205 increased the mRNA levels of key antioxidant transcription factors (e.g., Nrf1, Nfe2l2) and enzymes (e.g., Sod2, Prdx1, Prdx6 and Gclc). These findings suggest that MyoMed‐205 may improve antioxidant defense mechanisms, which likely contribute to protecting diaphragm muscle fibre structure and function under denervation stress.

Stress‐induced UPR activation contributes to maintaining muscle homeostasis under various stress states. Elevated oxidative stress and proteolysis contribute to myofibrillar protein damage, loss and muscle wasting [30]. Ultrastructural changes, including impaired sarcomere and Z‐disk structures, were observed in the diaphragm following denervation [8]. Accordingly, we observed increased UPR pathway activation, which indicates a cellular response against myofibrillar protein damage and loss after 12 h of UDD. Interestingly, MyoMed‐205 significantly enhanced UPR pathway activation at the transcriptional level by upregulating transcription factors (e.g., XBP1, ATF4) that regulate co‐chaperone (e.g., Bag3) and chaperone complexes (e.g., Cryab, Hspa5, Hspa8, Hspb8, Hsp90b1, Dnjb11, Cct5, etc.). MyoMed‐205 also upregulated the expression of genes involved in sarcomere integrity maintenance. Bag3 is known to facilitate the chaperone‐assisted selective autophagy (CASA) process for myofibrillar protein maintenance, and its downregulation impairs contractile function and protein turnover [31]. Bag3 can also interact with alpha B‐crystallin (i.e., Cryab) to regulate I‐band and Z‐disk integrity, while its regulation of Hsp70 chaperone activity protects against damaged protein aggregates and muscle homeostasis [32]. Similarly, chaperone co‐inducer BGP‐15 attenuates ventilation‐induced diaphragmatic dysfunction by activating Hsp72 and reducing myosin PTMs [33]. These findings indicate that MyoMed‐205–mediated protective effects might involve enhanced transcriptional activation of chaperones and UPR during UDD stress.

Muscle mass regulation crucially depends on the balance between protein synthesis and degradation. After 12 h of UDD, we observed comparable levels of atrophy across slow‐ and fast‐twitch fibres, despite a trend towards a slightly greater atrophy in Type IIb/x fibres, consistent with previous findings in similar durations (i.e., 24 h) of diaphragm paralysis [7]. Importantly, some studies also reported no changes in slow‐ or fast‐twitch fibre size at 24 h of UDD [5]. On the other hand, fibre type–specific responses seem more pronounced in long‐term UDD. Rodents subjected to several days (e.g., 3–14) of UDD exhibit predominant atrophy of fast‐twitch fibres, particularly of Type IIb/x, whereas slow‐twitch fibres, which are essential for basal respiratory function, appear less affected, unaltered or display transient hypertrophy [5, 8, 9, 12, 34]. However, the long‐term effects of UDD on diaphragm muscle fibres are intricate and remain not completely understood. Clinical observations illustrate this complexity as, in humans, prolonged UDD has been shown to induce progressive diaphragm fibre atrophy, with slow‐twitch fibres appearing to be more rapidly and severely affected than fast‐twitch fibres [35].

The decline in diaphragm fibre size after 12 h of UDD indicates a rapid shift towards a predominantly catabolic state. However, we simultaneously observed an increased expression of genes linked to protein synthesis and muscle growth (e.g., Myc, MAPK/ERK and mTORC1) following UDD, suggesting the activation of compensatory mechanisms to counteract muscle catabolism, in line with previous reports [10, 11]. Importantly, MyoMed‐205 prevented denervation‐induced diaphragm fibre atrophy and enhanced gene expression of pathways associated with RNA processing, ribosome biogenesis, protein synthesis and cell growth (e.g., Myc, Nr4a3, PI3K‐Akt–mTOR, eIF4e), which is consistent with the evidence that MuRF1 can directly target ribosomal components and translation factors [16, 17, 18].

We previously have shown that MyoMed‐205 increases Akt activation in the diaphragm under early UDD stress [6]. Herein, we further validated the MyoMed‐205–mediated activation of the Akt–mTOR pathway at the protein level within 12 h of UDD. Previous studies demonstrated that a murine MuRF1 gene knockout enhances Akt activation [24] and *de novo* protein synthesis. Thereby, MuRF1 KO mice seem less susceptible to muscle wasting [36]. Furthermore, MuRF1 can indirectly influence Myc activity by targeting Bin1, a negative regulator of Myc [18], which was found significantly upregulated in MyoMed‐205–treated rats. MyoMed‐205 also enhanced NR4A3 gene expression, which positively regulates mTORC1 signalling and contributes to exercise‐induced muscle hypertrophy [37]. Our data suggests that MyoMed‐205 activates anabolic pathways at the gene and protein levels that might contribute to counteract UDD‐associated diaphragm muscle atrophy.

Increased protein damage and breakdown also promote muscle wasting. In this study, we found a significant upregulation of inflammatory and proteolytic pathways (e.g., IL6‐JAK‐STAT3, TNFα/NFκB, ubiquitin‐proteasome system) in the diaphragm after 12 h of UDD. Noteworthy, animals treated with MyoMed‐205 showed relatively higher gene expression of MuRF1 (i.e., TRIM63) compared with DNV_VEH, although not statistically significant, and without affecting protein levels. This may suggest a compensatory cellular mechanism in an attempt to enhance MuRF1 expression and/or activity following denervation, consistent with previous studies [38]. This finding underscores the importance of dose–response optimization, as excessive MyoMed‐205 levels may disrupt muscle homeostasis and function, as we previously showed [6]. Conversely, we observed increased MuRF2 protein levels following 12 h of UDD, a response abrogated by MyoMed‐205, in line with previous studies suggesting that MuRF1 deletion/inhibition may downregulate MuRF2 expression, and that MyoMed‐205 may also affect MuRF2 [20]. This effect may compromise MuRF1‐MuRF2 heterodimerization and its cooperative role in myofibrillar protein ubiquitination [6, 21, 39]. Therefore, MyoMed‐205–mediated protection against diaphragm fibres contractile dysfunction and atrophy might involve the disruption of MuRF1‐MuRF2 cooperative role in myofibrillar breakdown.

Sustained cellular stress triggers myofibre apoptosis and diaphragm tissue remodelling, including intramuscular fat accumulation and fibrosis. Accordingly, we found upregulated transcription of adipogenic genes concomitantly with a higher number of ORO+ muscle cells with increased intracellular lipid content after 12 h of UDD. MyoMed‐205 alleviated these effects, likely through downregulation of key adipogenic genes (e.g., Adiponectin, Pparγ). This might involve MyoMed‐205 mediated blockade of MuRF2, known to suppress Pparγ expression/activity [40]. Furthermore, UDD enhanced gene expression linked to ECM collagen deposition and fibrosis, consistent with previous studies [13]. Notably, MyoMed‐205 suppressed the denervation‐induced ECM collagen deposition response at the transcriptional level. Previous experimental studies demonstrated that small‐molecule targeting MuRF1 mitigate cardiac fibrosis, improving contractile function during heart failure [22]. Our data indicate that MyoMed‐205 may contribute to the structural preservation of the diaphragm by mitigating denervation‐induced intramuscular lipid accumulation and ECM collagen deposition triggered by UDD.

## 5 Study Limitations

This is the first study to provide mechanistic insights into how the small‐molecule targeting MuRF1 (MyoMed‐205) protects against early denervation‐induced diaphragmatic dysfunction and atrophy in an experimental model. However, the following limitations must be considered: 1) These analyses were performed at a single time point (12 h), limiting assessment of temporal dynamics in small‐molecule targeting MuRF1 effects. 2) This study was limited to male rats treated with a single dosage (50 mg/kg bw), precluding assessment of potential sex‐specific differences or dose–response relationships in response to denervation and MyoMed‐205 treatment. 3) Our mechanistic findings rely primarily on transcriptomic and protein content analyses, which do not account for important regulatory mechanisms such as posttranscriptional and PTMs or changes in protein activity. 4) Cause‐and‐effect experiments were not feasible at this time to validate which mechanisms are crucial for small‐molecule targeting MuRF1‐mediated protection during early UDD. Future studies should employ multiomics analyses, assess different dosage ranges in both sexes and evaluate multiple time points to further comprehend the complex molecular mechanisms underlying small‐molecule targeting MuRF1‐mediated protection against denervation‐induced diaphragm weakness and wasting.

## 6 Conclusions

The small‐molecule targeting MuRF1, MyoMed‐205, protects against denervation‐induced diaphragm muscle contractile dysfunction and atrophy in rats undergoing 12 h of UDD [6]. In this study, our mechanistic findings reveal that MyoMed‐205 enhances the transcriptional mechanisms involved with the control of sarcomere structure and function, antioxidant defence, chaperone‐mediated UPR and muscle cell growth. MyoMed‐205 also attenuated intramuscular lipid accumulation and pro‐fibrotic responses triggered by UDD. These findings indicate MuRF1 as a promising therapeutic target for counteracting denervation‐induced diaphragm muscle contractile dysfunction and atrophy.

## Author Contributions

FR and ASM were involved in the study's conceptualization. FR conducted animal experiments, sample collection, functional, morphometric and molecular analyses. PRJ conducted bioinformatic analysis (RNA‐sequencing). FR and ASM performed data management and analysis. FR and ASM prepared the original manuscript. ASM and SL were involved in acquiring funding and project supervision. All authors were involved in the critical revision of the manuscript. All authors read and approved the final manuscript.

## Ethics Statement

All animal studies have been approved by the appropriate ethics committee (CEUA ICB USP protocols #8728030320 and #5143091020) and have therefore been performed following the ethical standards laid down in the 1964 Declaration of Helsinki and its later amendments.

## Conflicts of Interest

Siegfried Labeit reports a patent filing for MyoMed‐205 and further derivatives for its application to chronic muscle stress states (Patent Accession No WO2021023643A1). The other authors declare no conflicts of interest.

## Supporting information


**Data S1:** Supporting information.

**Figure S1:** Quality control analysis of the RNA samples isolated from the diaphragm for RNA‐seq‐based global gene expression profiling. (A) RNA ScreenTape quality control analysis and samples' RNA integrity number (RIN) scores. The four RNA samples of each experimental group with higher purity and integrity scores that were selected for RNA‐seq transcriptomic analysis are highlighted in red. (B) RNA‐seq quality control assessment of the number of genes detected, mitochondrial and ribosomal gene proportions. (C) RNA‐seq sample distances analysis representing the overall gene expression similarities or differences among the samples.

**Figure S2:** MyoMed‐205 upregulates RNA processing and transcription under 12 h of unilateral diaphragm denervation. (A) Heat map of genes associated with the Cajal Body. (B) Heat map of genes associated with the nuclear speck. (C) Heat map of genes associated with the spliceosome complex. (D) Heat map of genes associated with the transcription export complex.

**Figure S3:** Effects of unilateral denervation and MyoMed‐205 upon noncanonical signalling pathways involved with muscle cell growth and survival in the diaphragm. (A) Heat map of genes involved in KRAS signalling that were upregulated by denervation in the diaphragm. (B) Heat map of genes involved in KRAS signalling that were downregulated by denervation in the diaphragm. (C) Heat map of genes involved in androgen response that were upregulated by MyoMed‐205.

**Figure S4:** Denervation enhances inflammatory and immune responses in the diaphragm. (A) Heat map of genes associated with TNFα signalling via NFκB. (B) Heat map of genes associated with inflammatory response. (C) Heat map of genes associated with the complement system. (D) Heat map of genes associated with interferon gamma response.

**Figure S5:** Denervation enhances TGF‐β signalling in the diaphragm. Heat map of genes associated with the TGF‐β–signalling that were modulated following 12 h of unilateral diaphragm denervation.

**Table S1:** List of antibodies used for immunofluorescence and western blotting analysis. IF, immunofluorescence; WB, western blotting. Antibodies were diluted in a 5% BSA PBS solution during the immunofluorescent assay. During western blotting assays, antibodies were diluted in a 5% BSA or nonfat dry milk TBS‐T 0.1% solution.

**Table S2:** Quality control parameters of the RNA isolated from diaphragm muscles. sham, sham‐operated 12 h controls; DNV_VEH, denervation 12 h + vehicle treatment; DNV_205, denervation 12 h + MyoMed‐205 treatment; NA, not applicable; RIN, RNA integrity number.

## References

[1] M. J. Fogarty , C. B. Mantilla , and G. C. Sieck , “Breathing: Motor Control of Diaphragm Muscle,” Physiology 33 (2018): 113–126. 29412056 10.1152/physiol.00002.2018 PMC5899234

[2] F. D. McCool and G. E. Tzelepis , “Dysfunction of the Diaphragm,” New England Journal of Medicine 366 (2012): 932–942. 22397655 10.1056/NEJMra1007236

[3] H. M. Gransee , C. B. Mantilla , and G. C. Sieck , “Respiratory Muscle Plasticity,” Comprehensive Physiology 2 (2012): 1441–1462. 23798306 10.1002/cphy.c110050 PMC3962767

[4] G. C. Sieck and M. J. Fogarty , “Diaphragm Muscle: A Pump That Can Not Fail,” Physiological Reviews 105 (2025): 2589–2656, 10.1152/physrev.00043.2024. 40643074 PMC12363152

[5] C. Shindoh , W. Hida , H. Kurosawa , et al., “Effects of Unilateral Phrenic Nerve Denervation on Diaphragm Contractility in Rat,” Tohoku Journal of Experimental Medicine 173 (1994): 291–302. 7846681 10.1620/tjem.173.291

[6] F. Ribeiro , P. K. N. Alves , L. R. G. Bechara , J. C. B. Ferreira , S. Labeit , and A. S. Moriscot , “Small‐Molecule Inhibition of MuRF1 Prevents Early Disuse‐Induced Diaphragmatic Dysfunction and Atrophy,” International Journal of Molecular Sciences 24 (2023): 3637. 36835047 10.3390/ijms24043637 PMC9965746

[7] L. C. Gill , H. H. Ross , K. Z. Lee , et al., “Rapid Diaphragm Atrophy Following Cervical Spinal Cord Hemisection,” Respiratory Physiology & Neurobiology 192 (2014): 66–73. 24341999 10.1016/j.resp.2013.12.006 PMC4017782

[8] L. E. Gosselin , G. Brice , B. Carlson , Y. S. Prakash , and G. C. Sieck , “Changes in Satellite Cell Mitotic Activity During Acute Period of Unilateral Diaphragm Denervation,” Journal of Applied Physiology 77 (1994): 1128–1134. 7836114 10.1152/jappl.1994.77.3.1128

[9] B. Aravamudan , C. B. Mantilla , W. Z. Zhan , and G. C. Sieck , “Denervation Effects on Myonuclear Domain Size of Rat Diaphragm Fibers,” Journal of Applied Physiology 100 (2006): 1617–1622. 16410375 10.1152/japplphysiol.01277.2005

[10] H. M. Argadine , N. J. Hellyer , C. B. Mantilla , W. Z. Zhan , and G. C. Sieck , “The Effect of Denervation on Protein Synthesis and Degradation in Adult Rat Diaphragm Muscle,” Journal of Applied Physiology 107 (2009): 438–444. 19520837 10.1152/japplphysiol.91247.2008 PMC2724326

[11] H. M. Argadine , C. B. Mantilla , W. Z. Zhan , and G. C. Sieck , “Intracellular Signaling Pathways Regulating Net Protein Balance Following Diaphragm Muscle Denervation,” American Journal of Physiology‐Cell Physiology 300 (2011): 318–327. 10.1152/ajpcell.00172.2010 PMC3043627 21084642

[12] R. van der Pijl , J. Strom , S. Conijn , et al., “Titin‐Based Mechanosensing Modulates Muscle Hypertrophy,” Journal of Cachexia, Sarcopenia and Muscle 9 (2018): 947–961. 29978560 10.1002/jcsm.12319 PMC6204599

[13] L. E. Gosselin , G. C. Sieck , R. A. Aleff , D. A. Martinez , and A. C. Vailas , “Changes in Diaphragm Muscle Collagen Gene Expression After Acute Unilateral Denervation,” Journal of Applied Physiology 79 (1995): 1249–1254. 8567569 10.1152/jappl.1995.79.4.1249

[14] T. Centner , J. Yano , E. Kimura , et al., “Identification of Muscle Specific Ring Finger Proteins as Potential Regulators of the Titin Kinase Domain,” Journal of Molecular Biology 306 (2001): 717–726. 11243782 10.1006/jmbi.2001.4448

[15] S. C. Bodine , E. Latres , S. Baumhueter , et al., “Identification of Ubiquitin Ligases Required for Skeletal Muscle Atrophy,” Science (80‐) 294 (2001): 1704–1708. 10.1126/science.1065874 11679633

[16] S. H. Witt , H. Granzier , C. C. Witt , and S. Labeit , “MURF‐1 and MURF‐2 Target a Specific Subset of Myofibrillar Proteins Redundantly: Towards Understanding MURF‐Dependent Muscle Ubiquitination,” Journal of Molecular Biology 350 (2005): 713–722. 15967462 10.1016/j.jmb.2005.05.021

[17] C. C. Witt , S. H. Witt , S. Lerche , D. Labeit , W. Back , and S. Labeit , “Cooperative Control of Striated Muscle Mass and Metabolism by MuRF1 and MuRF2,” EMBO Journal 27 (2008): 350–360. 18157088 10.1038/sj.emboj.7601952 PMC2168395

[18] L. M. Baehr , D. C. Hughes , S. A. Lynch , et al., “Identification of the MuRF1 Skeletal Muscle Ubiquitylome Through Quantitative Proteomics,” Function 2 (2021): 1–18. 10.1093/function/zqab029 PMC8218097 34179788

[19] D. Peris‐Moreno , D. Taillandier , and C. Polge , “MuRF1/TRIM63, Master Regulator of Muscle Mass,” International Journal of Molecular Sciences 21 (2020): 6663. 32933049 10.3390/ijms21186663 PMC7555135

[20] T. S. Bowen , V. Adams , S. Werner , et al., “Small‐Molecule Inhibition of MuRF1 Attenuates Skeletal Muscle Atrophy and Dysfunction in Cardiac cachexia,” Journal of Cachexia, Sarcopenia and Muscle 8 (2017): 939–953. 28887874 10.1002/jcsm.12233 PMC5700443

[21] V. Adams , T. S. Bowen , S. Werner , et al., “Small‐Molecule‐Mediated Chemical Knock‐Down of MuRF1/MuRF2 and Attenuation of Diaphragm Dysfunction in Chronic Heart Failure,” Journal of Cachexia, Sarcopenia and Muscle 10 (2019): 1102–1115. 31140761 10.1002/jcsm.12448 PMC6818456

[22] V. Adams , A. Schauer , A. Augstein , et al., “Targeting MuRF1 by Small Molecules in a HFpEF rat Model Improves Myocardial Diastolic Function and Skeletal Muscle Contractility,” Journal of Cachexia, Sarcopenia and Muscle 13 (2022): 1565–1581. 35301823 10.1002/jcsm.12968 PMC9178400

[23] V. Adams , V. Gußen , S. Zozulya , et al., “Small‐Molecule Chemical Knockdown of MuRF1 in Melanoma Bearing Mice Attenuates Tumor Cachexia Associated Myopathy,” Cells 9 (2020): 2272. 33050629 10.3390/cells9102272 PMC7600862

[24] S. Labeit , S. Hirner , J. Bogomolovas , et al., “Regulation of Glucose Metabolism by MuRF1 and Treatment of Myopathy in Diabetic Mice With Small Molecules Targeting MuRF1,” International Journal of Molecular Sciences 22 (2021): 2225. 33672385 10.3390/ijms22042225 PMC7926706

[25] P. E. Hooijman , A. Beishuizen , C. C. Witt , et al., “Diaphragm Muscle Fiber Weakness and Ubiquitin–Proteasome Activation in Critically ill Patients,” American Journal of Respiratory and Critical Care Medicine 191 (2015): 1126–1138. 25760684 10.1164/rccm.201412-2214OC PMC4451621

[26] J. Liu , Y. Chen , D. Han , and M. Huang , “Inhibition of the Expression of TRIM63 Alleviates Ventilator‐Induced Diaphragmatic Dysfunction by Modulating the PPARα/PGC‐1α Pathway,” Mitochondrion 83 (2025): 102025. 40049543 10.1016/j.mito.2025.102025

[27] S. Matecki , H. Dridi , B. Jung , et al., “Leaky Ryanodine Receptors Contribute to Diaphragmatic Weakness During Mechanical Ventilation,” Proceedings of the National Academy of Sciences of the United States of America 113 (2016): 9069–9074. 27457930 10.1073/pnas.1609707113 PMC4987795

[28] R. Kiewitz , C. Acklin , B. W. Schäfer , et al., “Ca2+−Dependent Interaction of S100A1 With the Sarcoplasmic Reticulum Ca2+‐ATPase2a and Phospholamban in the Human Heart,” Biochemical and Biophysical Research Communications 306 (2003): 550–557. 12804600 10.1016/s0006-291x(03)00987-2

[29] P. Most , J. Bernotat , P. Ehlermann , et al., “S100A1: A Regulator of Myocardial Contractility,” Proceedings of the National Academy of Sciences 98 (2001): 13889–13894. 10.1073/pnas.241393598 PMC61137 11717446

[30] R. A. Shanely , M. A. Zergeroglu , S. L. Lennon , et al., “Mechanical Ventilation–Induced Diaphragmatic Atrophy Is Associated With Oxidative Injury and Increased Proteolytic Activity,” American Journal of Respiratory and Critical Care Medicine 166 (2002): 1369–1374. 12421745 10.1164/rccm.200202-088OC

[31] T. G. Martin , V. D. Myers , P. Dubey , et al., “Cardiomyocyte Contractile Impairment in Heart Failure Results From Reduced BAG3‐Mediated Sarcomeric Protein Turnover,” Nature Communications 12 (2021): 2942. 10.1038/s41467-021-23272-z PMC8134551 34011988

[32] M. Meister‐Broekema , R. Freilich , C. Jagadeesan , et al., “Myopathy Associated BAG3 Mutations Lead to Protein Aggregation by Stalling Hsp70 Networks,” Nature Communications 9 (2018): 5342. 10.1038/s41467-018-07718-5 PMC6297355 30559338

[33] H. Salah , M. Li , N. Cacciani , et al., “The Chaperone Co‐Inducer BGP‐15 Alleviates Ventilation‐Induced Diaphragm Dysfunction,” Science Translational Medicine 8 (2016): 350ra103. 10.1126/scitranslmed.aaf7099 27488897

[34] P. C. Geiger , M. J. Cody , R. L. Macken , M. E. Bayrd , and G. C. Sieck , “Effect of Unilateral Denervation on Maximum Specific Force in Rat Diaphragm Muscle Fibers,” Journal of Applied Physiology 90 (2001): 1196–1204. 11247914 10.1152/jappl.2001.90.4.1196

[35] W. N. Welvaart , M. A. Paul , H. W. H. van Hees , et al., “Diaphragm Muscle Fiber Function and Structure in Humans With Hemidiaphragm Paralysis,” American Journal of Physiology. Lung Cellular and Molecular Physiology 301 (2011): 228–235. 10.1152/ajplung.00040.2011 PMC7938767 21622847

[36] S. Koyama , S. Hata , C. C. Witt , et al., “Muscle RING‐Finger Protein‐1 (MuRF1) as a Connector of Muscle Energy Metabolism and Protein Synthesis,” Journal of Molecular Biology 376 (2008): 1224–1236. 18222470 10.1016/j.jmb.2007.11.049

[37] N. J. Pillon , B. M. Gabriel , L. Dollet , et al., “Transcriptomic Profiling of Skeletal Muscle Adaptations to Exercise and Inactivity,” Nature Communications 11 (2020): 470. 10.1038/s41467-019-13869-w PMC6981202 31980607

[38] J. D. Furlow , M. L. Watson , D. S. Waddell , et al., “Altered Gene Expression Patterns in Muscle Ring Finger 1 Null Mice During Denervation‐ and Dexamethasone‐Induced Muscle Atrophy,” Physiological Genomics 45 (2013): 1168–1185. 24130153 10.1152/physiolgenomics.00022.2013 PMC3882710

[39] T. Nguyen , T. S. Bowen , A. Augstein , et al., “Expression of MuRF1 or MuRF2 is Essential for the Induction of Skeletal Muscle Atrophy and Dysfunction in a Murine Pulmonary Hypertension Model,” Skeletal Muscle 10 (2020): 1–10. 32340625 10.1186/s13395-020-00229-2 PMC7184701

[40] J. He , M. T. Quintana , J. Sullivan , et al., “MuRF2 Regulates PPARγ1 Activity to Protect Against Diabetic Cardiomyopathy and Enhance Weight Gain Induced by a High Fat Diet,” Cardiovascular Diabetology 14 (2015): 97. 26242235 10.1186/s12933-015-0252-x PMC4526192

