# Supplementary figures and images for: Small‐Molecule Targeting MuRF1 Protects Against Denervation‐Induced Diaphragmatic Dysfunction: Underlying Molecular Mechanisms
Source: J Cachexia Sarcopenia Muscle. 2025 Nov 16;16(6):e70119. doi: 10.1002/jcsm.70119 (PMC12620420; doi:10.1002/jcsm.70119)

**A**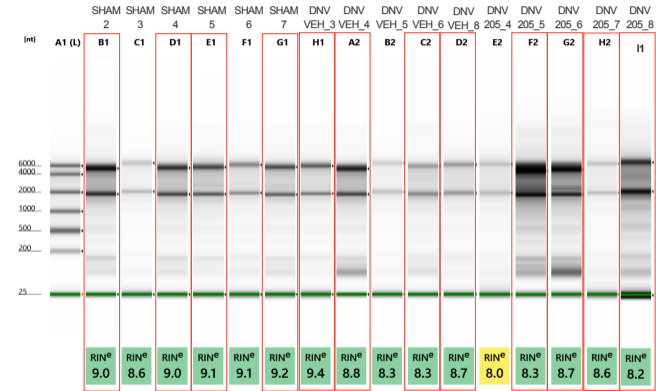

Default image (Contrast 100%)

**B**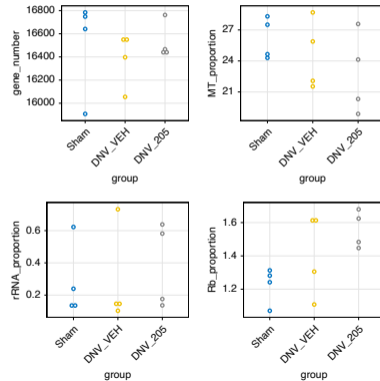**C**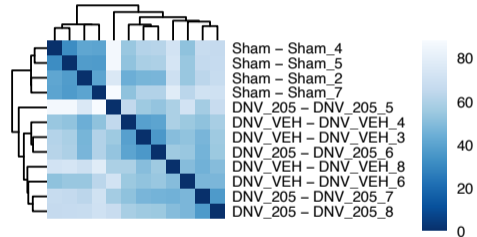

Supplement: Supplementary file 2 — Figure S1: Quality control analysis of the RNA samples isolated from the diaphragm for RNA‐seq‐based global gene expression profiling. (A) RNA ScreenTape quality control analysis and samples' RNA integrity number (RIN) scores. The four RNA samples of each experimental group with higher purity and integrity scores that were selected for RNA‐seq transcriptomic analysis are highlighted in red. (B) RNA‐seq quality control assessment of the number of genes detected, mitochondrial and ribosomal gene proportions. (C) RNA‐seq sample distances analysis representing the overall gene expression similarities or differences among the samples. [file JCSM-16-e70119-s006.pdf]

A

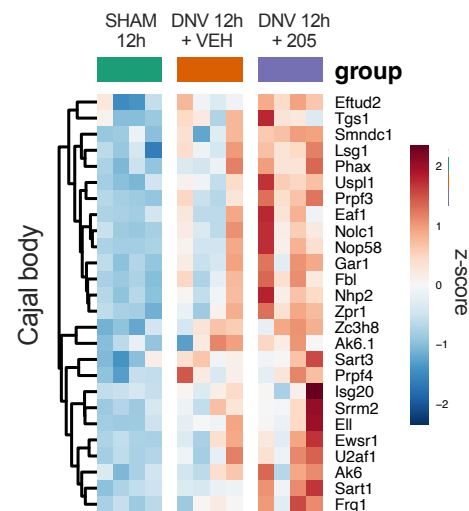

B

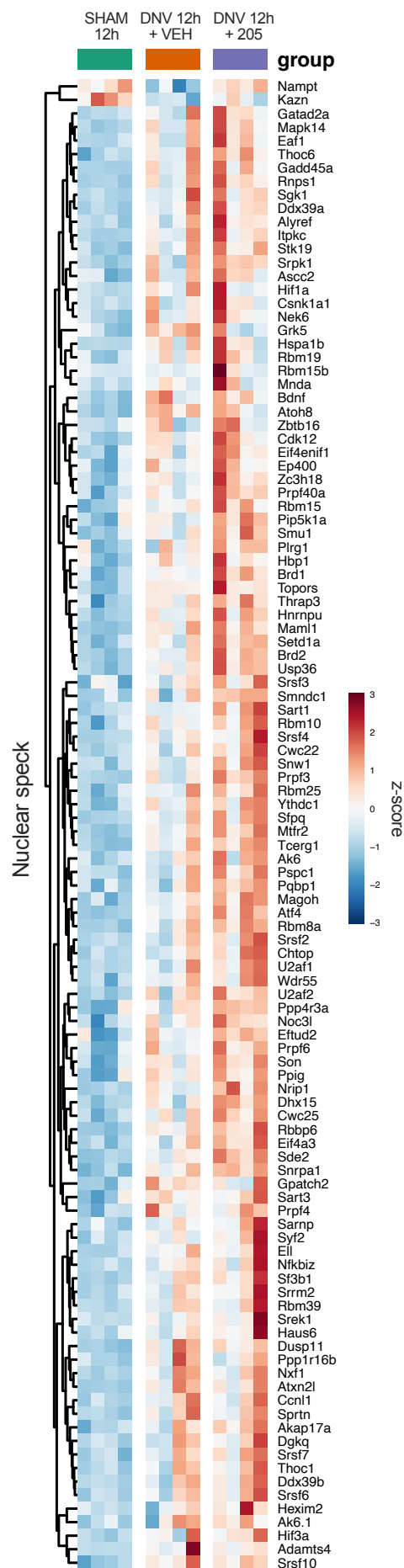

C

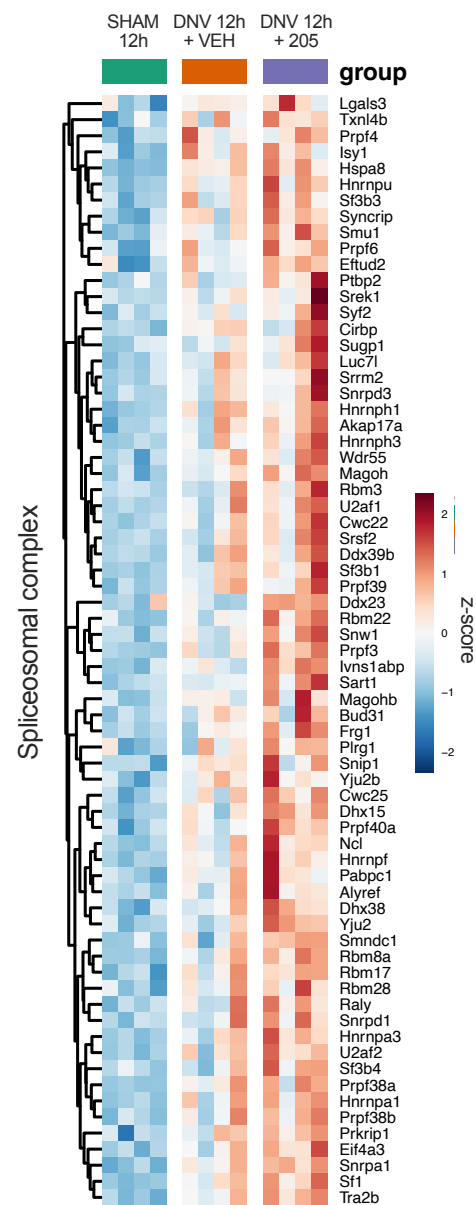

D

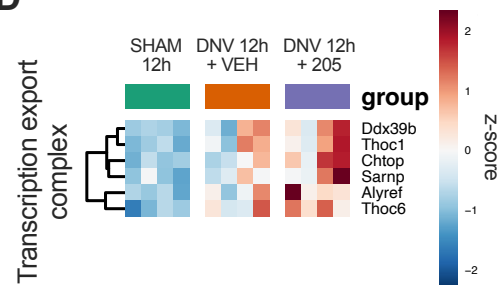

Supplement: Supplementary file 3 — Figure S2: MyoMed‐205 upregulates RNA processing and transcription under 12 h of unilateral diaphragm denervation. (A) Heat map of genes associated with the Cajal Body. (B) Heat map of genes associated with the nuclear speck. (C) Heat map of genes associated with the spliceosome complex. (D) Heat map of genes associated with the transcription export complex. [file JCSM-16-e70119-s002.pdf]

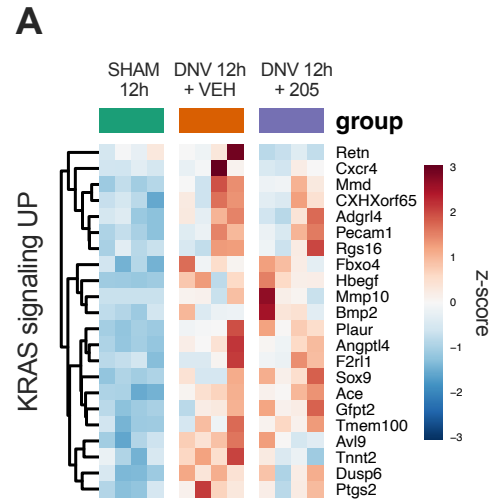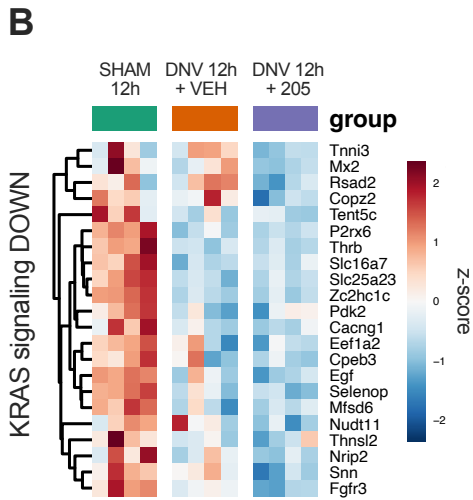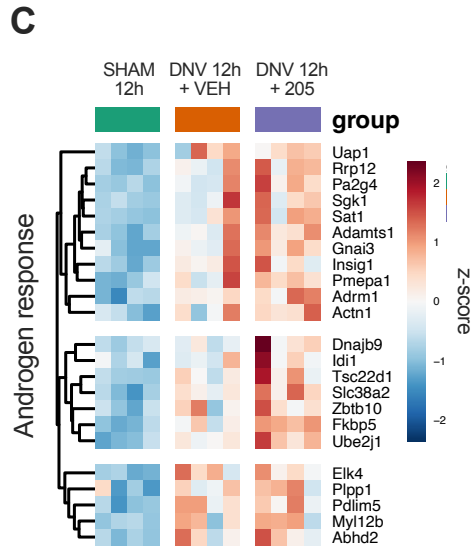

Supplement: Supplementary file 4 — Figure S3: Effects of unilateral denervation and MyoMed‐205 upon noncanonical signalling pathways involved with muscle cell growth and survival in the diaphragm. (A) Heat map of genes involved in KRAS signalling that were upregulated by denervation in the diaphragm. (B) Heat map of genes involved in KRAS signalling that were downregulated by denervation in the diaphragm. (C) Heat map of genes involved in androgen response that were upregulated by MyoMed‐205. [file JCSM-16-e70119-s008.pdf]

A

TNF $\alpha$  signaling via NF $\kappa$ B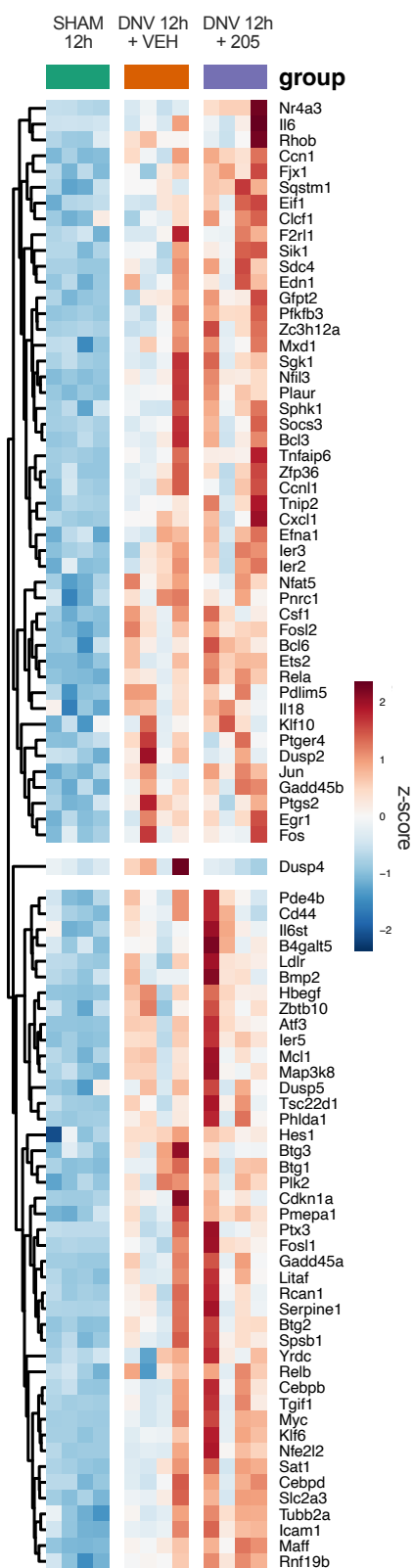

B

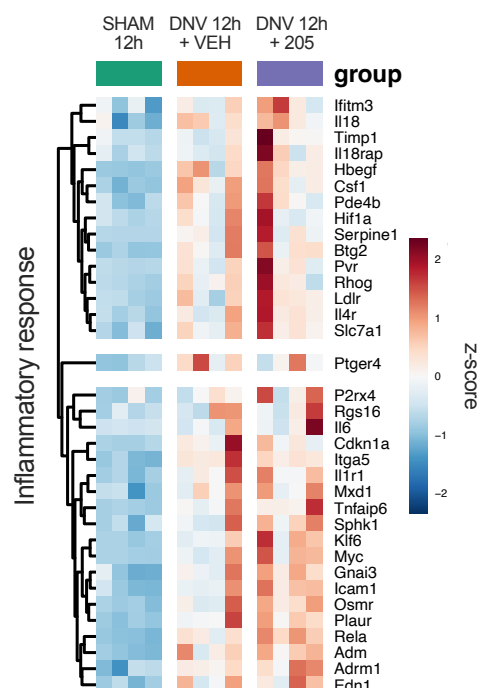

D

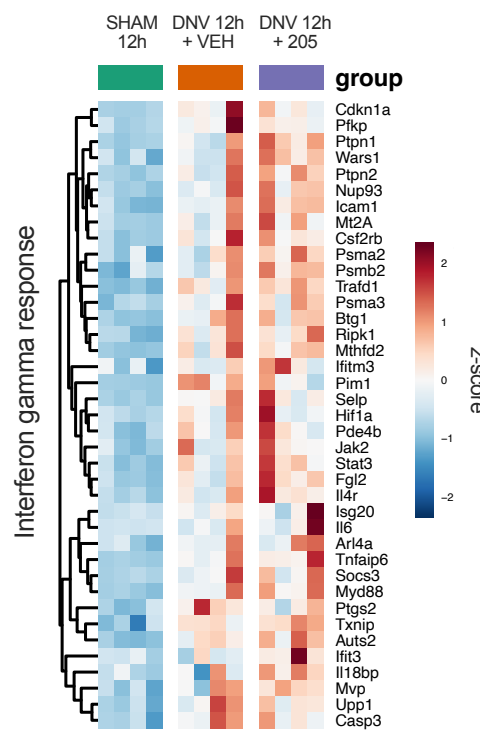

C

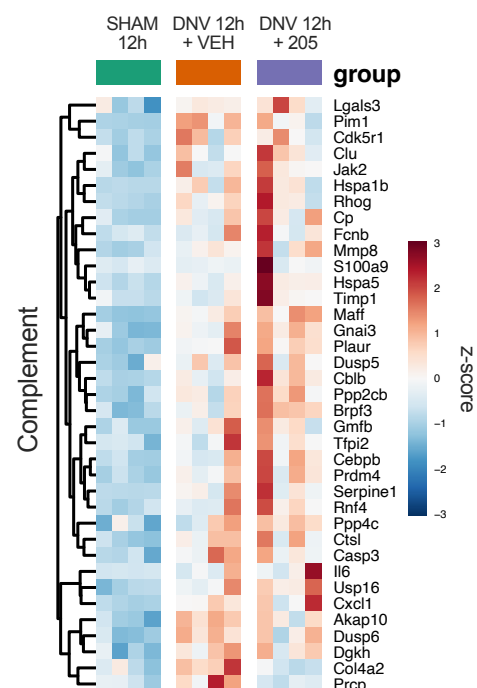

Supplement: Supplementary file 5 — Figure S4: Denervation enhances inflammatory and immune responses in the diaphragm. (A) Heat map of genes associated with TNFα signalling via NFκB. (B) Heat map of genes associated with inflammatory response. (C) Heat map of genes associated with the complement system. (D) Heat map of genes associated with interferon gamma response. [file JCSM-16-e70119-s003.pdf]
