# Supplementary material for: Small‐Molecule Targeting MuRF1 Protects Against Denervation‐Induced Diaphragmatic Dysfunction: Underlying Molecular Mechanisms
Source: J Cachexia Sarcopenia Muscle. 2025 Nov 16;16(6):e70119. doi: 10.1002/jcsm.70119 (PMC12620420; doi:10.1002/jcsm.70119)
Supplement: Supplementary file 6 — Figure S5: Denervation enhances TGF‐β signalling in the diaphragm. Heat map of genes associated with the TGF‐β–signalling that were modulated following 12 h of unilateral diaphragm denervation. [file JCSM-16-e70119-s005.pdf]

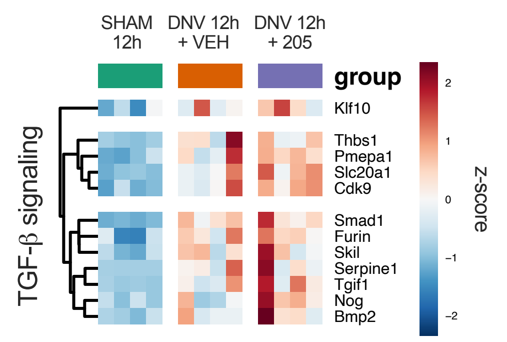
SHAM
12h
DNV 12h
+ VEH
DNV 12h
+ 205
group
TGF-β signaling
Klf10
Thbs1
Pmepa1
Slc20a1
Cdk9
Smad1
Furin
Skil
Serpine1
Tgif1
Nog
Bmp2
z-score
2
1
0
−1
−2
